# Subterranean environments contribute to three‐quarters of classified ecosystem services

**DOI:** 10.1002/brv.70137

**Published:** 2026-02-10

**Authors:** Stefano Mammola, David Brankovits, Tiziana Di Lorenzo, Isabel R. Amorim, Raluca Ioana Bancila, Adrià Bellvert, Enrico Bernard, Anna Blomberg, Paulo A.V. Borges, Martina Cappelletti, Rodrigo Lopes Ferreira, Rosalina Gabriel, Diana M. P. Galassi, Laura Garzoli, Vasilis Gerovasileiou, Grant C. Hose, Kathryn L. Korbel, Simone Martino, Ana Z Miller, Nataša Mori, Veronica Nanni, Giuseppe Nicolosi, Mattia Saccò, Troy S. Sakihara, Marconi Souza Silva, Anne E. Tamalavage, Maja Zagmajster, Efraín Chávez, Christian Griebler, Pedro Cardoso, Alejandro Martínez

**Affiliations:** ^1^ Molecular Ecology Group (MEG) Water Research Institute, National Research Council of Italy (CNR‐IRSA) Corso Tonolli 50 Verbania 28922 Italy; ^2^ Finnish Museum of Natural History University of Helsinki Pohjoinen Rautatiekatu 13 Helsinki 00100 Finland; ^3^ NBFC, National Biodiversity Future Center Piazza Marina 61 Palermo 90133 Italy; ^4^ Research Institute on Terrestrial Ecosystems, National Research Council (CNR‐IRET) Via Madonna del Piano 10, 50019 Sesto Fiorentino Florence Italy; ^5^ “Emil Racoviţă” Institute of Speleology Cluj‐Napoca Department Str. Clinicilor, Nr. 5‐7 Cluj‐Napoca 400006 Romania; ^6^ Centre for Ecology, Evolution and Environmental Changes & CHANGE – Global Change and Sustainability Institute and Departamento de Biologia Animal, Faculdade de Ciências, Universidade de Lisboa Campo Grande Lisbon 1749‐016 Portugal; ^7^ University of the Azores, CE3C—Centre for Ecology, Evolution and Environmental Changes, Azorean Biodiversity Group, CHANGE —Global Change and Sustainability Institute Rua Capitão João d'Ávila, Pico da Urze, 9700‐042 Angra do Heroísmo Azores Portugal; ^8^ IUCN SSC Atlantic Islands Invertebrate Specialist Group Angra do Heroísmo Azores 9700‐042 Portugal; ^9^ “Emil Racoviţă” Institute of Speleology Calea 13 Septembrie, Nr. 13 Bucharest 050711 Romania; ^10^ Laboratório de Ciência em Biodiversidade, Department of Ecology and Conservation Institute of Natural Sciences, Federal University of Lavras Lavras Minas Gerais 37200900 Brazil; ^11^ IUCN SSC Species Monitoring Specialist Group 9700‐042 Angra do Heroísmo Azores Portugal; ^12^ Department of Pharmacy and Biotechnology University of Bologna Via Irnerio 42 Bologna 40126 Italy; ^13^ La Venta Geographic Explorations Association Via Priamo Tron 35/F Treviso 31100 Italy; ^14^ Center for Studies in Subterranean Biology, Department of Ecology and Conservation Institute of Natural Sciences, Federal University of Lavras Lavras Minas Gerais 37200900 Brazil; ^15^ Department of Life, Health & Environmental Sciences University of L'Aquila Via Vetoio, Coppito L'Aquila 67100 Italy; ^16^ Department of Environment, Faculty of Environment Ionian University M. Minotou‐Giannopoulou str. Panagoula Zakynthos 29100 Greece; ^17^ Institute of Marine Biology, Biotechnology and Aquaculture (IMBBC), Hellenic Centre for Marine Research (HCMR) P.O. Box 2214 Heraklion 71003 Greece; ^18^ School of Natural Sciences, Macquarie University Sydney New South Wales 2109 Australia; ^19^ The James Hutton Institute Craigiebuckler Aberdeen Scotland AB15 8QH UK; ^20^ BIOGEOCOM ‐ Instituto de Recursos Naturales y Agrobiología de Sevilla (IRNAS‐CSIC) Avenida Reina Mercedes 10 Seville 41012 Spain; ^21^ HERCULES Lab University of Évora Largo Marquês de Marialva 8 Évora 7000‐809 Portugal; ^22^ Department of Organisms and Ecosystem Research National institute of Biology Večna pot 121 Ljubljana 1000 Slovenia; ^23^ Department of Biological, Geological and Environmental Sciences, Section Animal Biology University of Catania Catania 95124 Italy; ^24^ Subterranean Research and Groundwater Ecology (SuRGE) Group, Trace and Environmental DNA (TrEnD) Lab School of Molecular and Life Sciences, Curtin University Perth, Kent Street Bentley WA 6102 Australia; ^25^ Department of Chemistry, Life Sciences and Environmental Sustainability University of Parma Parco Area delle Scienze 11/A Parma 43124 Italy; ^26^ Laboratoire de Biologie des Organismes et des Écosystèmes Aquatiques‐BOREA. Muséum national d'Histoire naturelle SU, CNRS, IRD UA, 43 rue Cuvier CP 26 75231 Paris F‐75005 France; ^27^ Department of Land and Natural Resources, Division of Aquatic Resources 75 Aupuni Street, Room 204 Hilo Hawai'i 96720 USA; ^28^ Smithsonian Environmental Research Center Edgewater Maryland USA; ^29^ Biotechnical Faculty, Department of Biology, Subterranean Biology Lab (SubBioLab) University of Ljubljana Jamnikarjeva 101 Ljubljana 1000 Slovenia; ^30^ Unidad Multidisciplinaria de Docencia e Investigación Sisal, Facultad de Ciencias, Universidad Nacional Autónoma de México Puerto de Abrigo S/N Sisal Yucatán 97365 México; ^31^ Secretaria de Ciencia, Humanidades, Tecnología e Innovación Avenida Insurgentes Sur 1582, Benito Juárez Mexico City 03940 Mexico; ^32^ Department for Functional and Evolutionary Ecology University of Vienna Djerassiplatz 1 Vienna 1030 Austria

**Keywords:** groundwater, hypogean, nature value, drinking water, food production, biotechnology, geothermal energy, sustainability, ecotourism, cultural heritage

## Abstract

Beneath the Earth's surface lies a network of interconnected caves, voids, and systems of fissures forming in rocks of sedimentary, igneous, or metamorphic origin. Although largely inaccessible to humans, this hidden realm supports and regulates services critical to ecological health and human well‐being. Subterranean ecosystems are integral to major biogeochemical cycles, sustain diverse surface habitats, and serve as the primary source of irrigation and drinking water. They also offer non‐material benefits, including scientific discovery, education, and cultural practices. Yet, these contributions often go unrecognised, partly due to the lack of a unified synthesis of ecosystem services across terrestrial, freshwater, and marine subterranean compartments. This gap limits effective communication of their value to scientists, practitioners, and the public. Through a systematic expert‐based review, we show that subterranean ecosystems contribute to up to 75% of classified ecosystem services. Notably, many of these contributions are described only qualitatively, lacking numerical or economic quantification. Next, we list examples of the main ecosystem services provided by subterranean systems to offer a global overview of their multifaceted value and vulnerability to environmental change. We believe this synthesis provides researchers and practitioners with concrete examples to communicate more effectively the importance of subterranean ecosystems to diverse audiences.

## I. INTRODUCTION

Whether engaging in high‐stakes discussions with policymakers or navigating casual conversations at social gatherings, scientists studying subterranean biodiversity may find themselves in the uncomfortable position of defending the very essence of their work. Questions like, ‘Why waste your time in a muddy cave to count tiny beetles?’, ‘Are we really worried about some blind shrimp no one's ever seen?’ or ‘What's next—national parks for glow‐in‐the‐dark worms?’ are all too common. They reflect a deep misunderstanding of the hidden world beneath our feet, the fragile ecosystems it sustains, and the profound influence it has on the surface environments where humans live.

Studying ‘unremarkable’ species thriving beneath the Earth's surface might seem like an indulgent pursuit, far removed from the pressing concerns of modern life. After all, how could the presence of a whitish shrimp in a remote cave pond possibly contribute to global challenges such as economic growth, public health, or technological development? Far from trivial, these discussions reflect a broader struggle to spotlight the invisible services provided by nature (Rieb *et al*., 2017). The challenge, then, is not merely defending one's research but broadening collective understanding of biodiversity's essential functions: its intrinsic value and its critical role in maintaining a healthy, habitable planet (Loreau *et al*., 2021). The public cannot grasp what is at risk if scientists fail to communicate these values (Bekessy *et al*., 2018).

When the concept of ecosystem services gained momentum after 1997 (Costanza *et al*., 1997), it offered biodiversity scientists a powerful framework to articulate the societal relevance of their work (Lele *et al*., 2013). Ecosystem services encompass all the functions and products of ecosystems that benefit humans and contribute to societal welfare. Initially conceived as a metaphor (Norgaard, 2010), the concept evolved rapidly into a robust research agenda focused on cataloguing, quantifying, and mapping humanity's reliance on nature (Lele *et al*., 2013; Benra *et al*., 2024; Chaplin‐Kramer *et al*., 2025). For example, ecosystem services are frequently categorised into: provisioning services (e.g. food, water), regulation and maintenance services (e.g. climate regulation, pollination, air and water quality), and cultural services (e.g. recreational, traditional practices and spiritual well‐being) (Haines‐Young & Potschin‐Young, 2018). Many of these services also can be measured economically. This reflects the need to highlight the value of services that are, in part, subjective and difficult to perceive outside of academic contexts (Chee, 2004; Brander *et al*., 2024).

While the quantification of ecosystem services has occupied the research community for decades, knowledge remains incomplete for subterranean ecosystems. Subterranean ecosystems are globally distributed and vary widely in extent and type of matrix. Following the function‐based classification of Earth's ecosystems (Keith *et al*., 2020, 2022), we considered herein ecosystems belonging to these biomes in terrestrial, freshwater, and marine domains: ‘Subterranean’ (S) [including the ‘Subterranean lithic’ (S1) and ‘Anthropogenic subterranean voids’ (S2) biomes], ‘Subterranean‐freshwater’ (SF) [including the ‘Subterranean freshwater’ (SF1) and ‘Anthropogenic subterranean freshwater’ (SF2) biomes], and ‘Subterranean tidal’ (SM1). These include various types of caves (e.g. aerobic caves, lava tubes, volcanic pits, anchialine caves, sea caves) and other voids (e.g. fissure systems, deep scree strata), groundwater ecosystems and their ecotones (e.g. aquifers, underground streams, ponds, lakes, subterranean estuaries, anchialine pools, sinkholes, cenotes, blueholes, springs, hyporheic systems), as well as anthropogenic subterranean voids (e.g. mines, underground bunkers and tunnels, water pipes, subterranean canals, wells). These subterranean voids can vary greatly in depth, from very shallow layers in close contact with soils (Culver & Pipan, 2014) to several kilometres below the Earth's surface, reaching the so‐called endolithic systems (S1.2) (Pedersen, 2000; Edwards, Becker & Colwell, 2012; Keith *et al*., 2020).

**Fig. 1:**
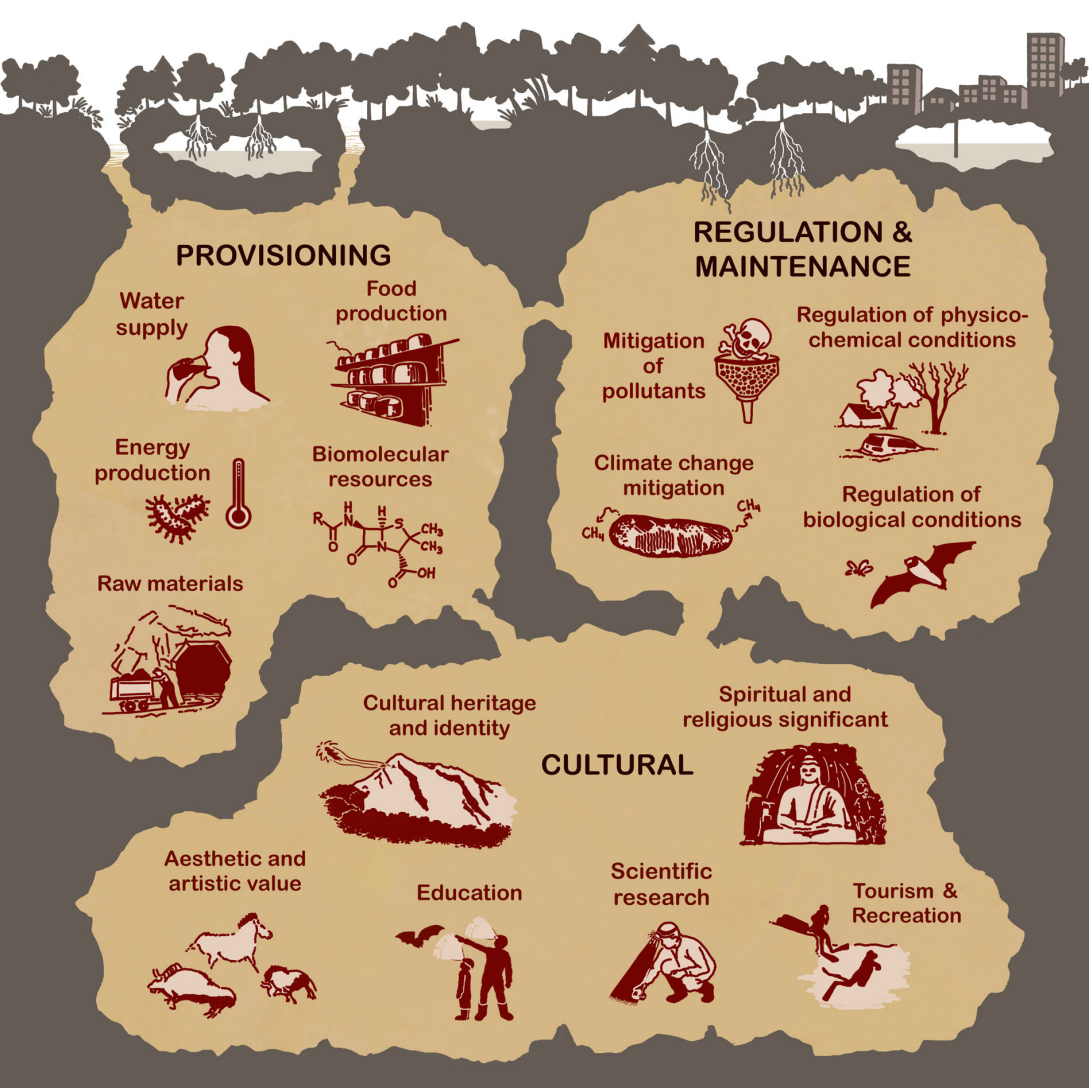
A visual summary of the main services associated with subterranean ecosystems. Original illustration by Jagoba Malumbres‐Olarte.

Despite their hidden nature, subterranean ecosystems provide and regulate services that are as critical to human well‐being and ecological health as those in surface ecosystems (Fig. 1). The benefits derived from subterranean ecosystems are remarkably diverse, with direct and indirect links to essential functions such as freshwater provisioning, food production, and the regulation of diverse biogeochemical and physical processes (Chapelle, 2000; Siebert *et al*., 2010; Griebler & Avramov, 2015; Gleeson *et al*., 2020). Subterranean ecosystems also contribute to essential ‘non‐material’ values, including scientific research and inspiration (Mammola, 2019; Mammola *et al*., 2020; Hesselberg, 2023), ecotourism (Chiarini, Duckeck & De Waele, 2022; Piano *et al*., 2024), aesthetic appreciation (Gleeson, 2024; Mammola *et al*., 2025), and cultural practices (Bertini, 2010; Moyes, 2012).

The questions driving this review are straightforward yet important: what services do subterranean ecosystems provide, and how many of these have been quantified to some extent? Answering these questions is urgent because, to paraphrase the common adage, ‘you can't manage what you can't see and measure’. Currently, information on the benefits provided by subterranean ecosystems is scattered across numerous sources, many of which remain inaccessible to the public. A handful of recent reviews have compiled subsets of these services for specific subterranean ecosystems, especially groundwaters (Herman, Culver & Salzman, 2001; Griebler & Avramov, 2015; Griebler, Avramov & Hose, 2019; Iliopoulos & Damigos, 2024; Charchousi, Goula & Papadopoulou, 2025), or species (e.g. bats; Medellin, Wiederholt & Lopez‐Hoffman, 2017). Yet, a comprehensive scheme that unifies all services across terrestrial, freshwater, and marine subterranean compartments is still lacking. Moreover, integrating quantitative rigor into this mapping exercise could enhance the perceived importance of these services and help establish connections to the eco‐evolutionary processes that sustain them. Such an understanding could shift the narrative from viewing subterranean ecosystems merely as sources of water, geothermal energy, and minerals to recognising their broader ecological value. This, in turn, would reinforce the importance of even partial data in designing conservation strategies that prioritise ecosystem functions over isolated species or habitats (Mammola *et al*., 2024).

## II. MAPPING OF SUBTERRANEAN ECOSYSTEM SERVICES

To map subterranean ecosystem services, we used the Common International Classification of Ecosystem Services (CICES Version 5.1). CICES is a classification scheme designed to measure, account for, and assess ecosystem services (Haines‐Young & Potschin‐Young, 2018). CICES defines ecosystem services as the *contributions that ecosystems make to human well‐being. […] It focuses on the ‘final’ outputs of ecosystems and seeks to identify the materials and properties of ecological systems that can be used by people in beneficial ways* (Haines‐Young & Potschin‐Young, 2018, p. 2). The services are categorised into three main Sections (Provisioning, Regulation & Maintenance, and Cultural services) and two broad types within each section (biotic and abiotic), with further breakdowns into levels of Division, Group, and Class. Conveniently, CICES is interoperable with other ecosystem service classification systems by providing equivalency across various schemes. CICES lists 90 primary services (63 biotic and 27 abiotic). Using this backbone classification, we assessed whether subterranean ecosystems contribute to the various ecosystem services listed in CICES based on our expert knowledge and the literature. To strengthen our evaluation, we conducted a literature review for each service to assess quantitative estimates of the services provided by subterranean ecosystems.

According to our mapping exercise (see online Supporting Information, Appendix S1), subterranean ecosystems contribute to up to 75% (68 out of 90) of the ecosystem services classified by CICES. This contribution is higher than the estimations for ecosystem services provided by grasslands (36%) (Richter *et al*., 2021), urban water bodies (43%) (Jakubiak & Chmielowski, 2020), mangrove ecosystems (33% of biotic services) (Mukherjee *et al*., 2014), or vineyards (64%) (Winkler, Viers & Nicholas, 2017). When considering specific systems, terrestrial, freshwater, and saltwater subterranean compartments contribute 48%, 57%, and 54% of the services classified by CICES, respectively.

**Fig. 2:**
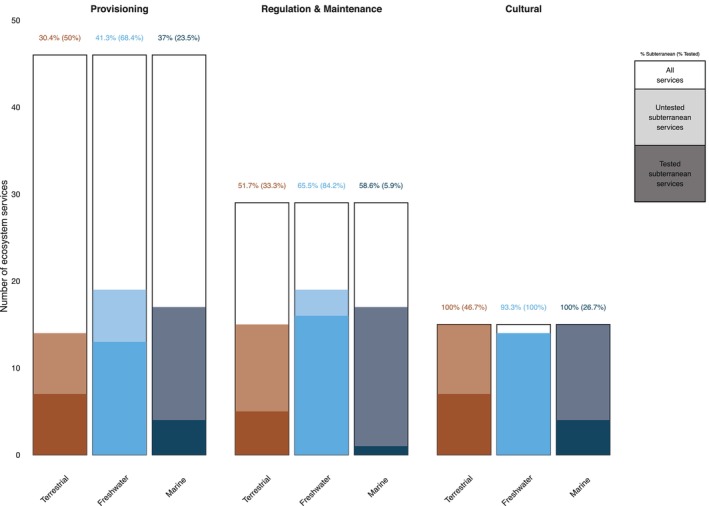
The number of Provisioning, Regulation & Maintenance, and Cultural services provided by terrestrial, freshwater, and marine subterranean ecosystems (coloured bars) compared to the total services mapped by the Common International Classification of Ecosystem Services (white bars). Darker shades indicate the fraction of subterranean services that have been quantitatively assessed in at least one study.

**Fig. 3:**
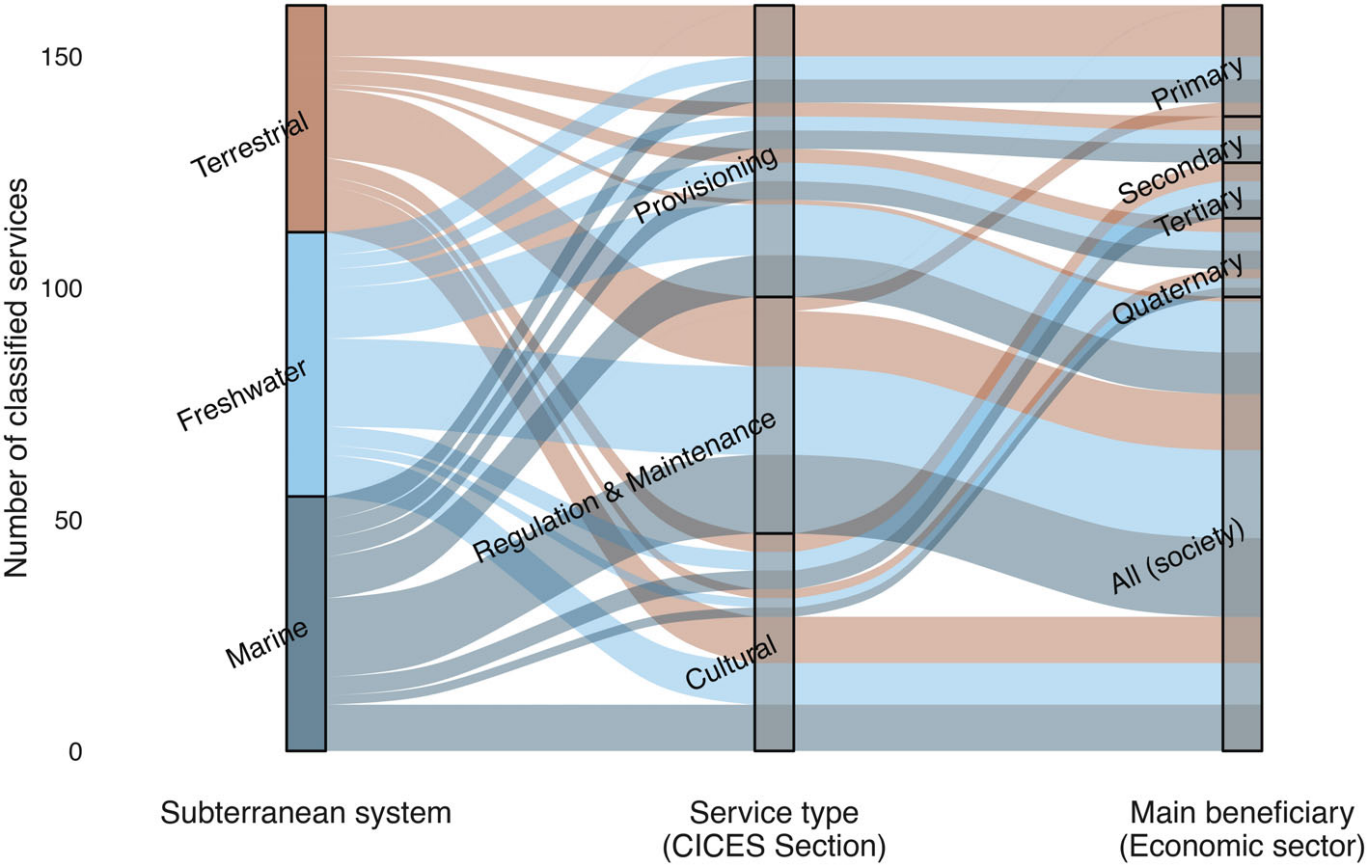
Importance of ecosystem services provided by terrestrial, freshwater, and marine subterranean ecosystems to socio‐economic sectors (primary: resource extraction; secondary: manufacturing; tertiary: services; quaternary: knowledge‐based activities). ‘All society’ represents services with multiple benefits not limited to a single sector.

Of the 68 matching services, between one third and a half have been tested/measured numerically (Fig. 2), primarily by local case studies. Most of the identified services benefit society at large, although some services appear to be most important for specific economic sectors (Fig. 3). Groundwaters, particularly freshwater systems, dominate in the percentage of quantified ecosystem services. This is likely due both to their accessibility and measurability compared to terrestrial and marine systems and the crucial importance of groundwater for drinking and irrigation. Indeed, human settlements are often located where there is access to aquifers through springs, caves, wells and boreholes. These features allow for direct sampling and regular monitoring. In comparison, terrestrial and marine subterranean ecosystems are less accessible, often requiring specialised and costly technologies for exploration (Kreamer *et al*., 2021; Mammola *et al*., 2021; Navarro‐Barranco *et al*., 2023).

## III. PROVISIONING SERVICES

Provisioning ecosystem services are the tangible goods and resources that ecosystems provide to humans (Haines‐Young & Potschin‐Young, 2018). These services are the direct products we obtain from nature, such as fresh water, food, raw materials, medicinal resources, and energy. Subterranean ecosystems contribute to as many as 63% of the provisioning ecosystem services classified by CICES.

### (1) Water supply

**Fig. 4:**
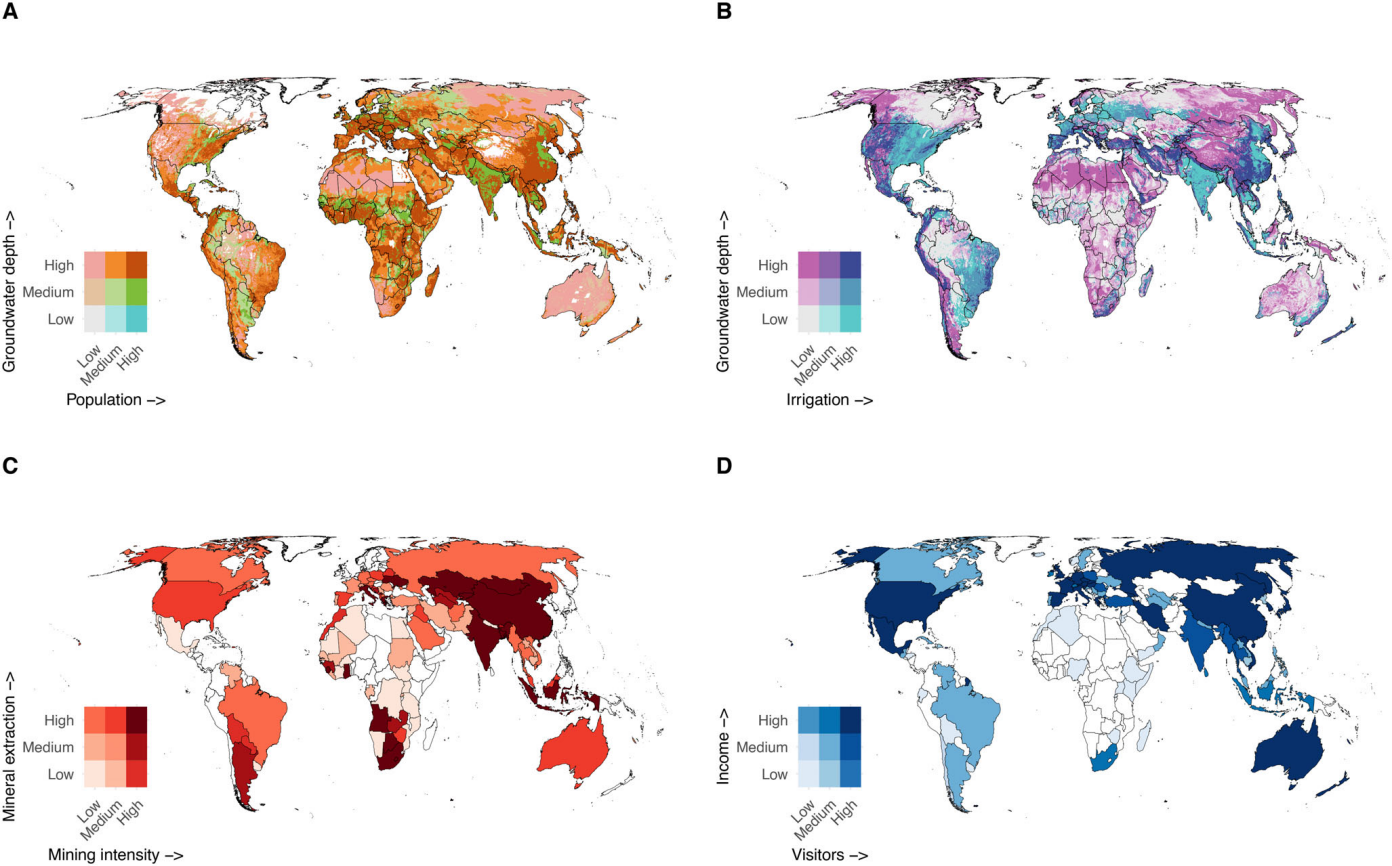
Global mapping of major subterranean ecosystem services based on proxy variables. (A) Groundwater depth and human population density, illustrating potential hotspots where there will be pressure in terms of groundwater extraction. Pink areas indicate regions where groundwater is deeply underground and difficult to access, with low population densities. Dark orange areas represent regions where groundwater is also difficult to access but have high population densities. (B) Groundwater depth and irrigation intensity, again illustrating potential hotspots where there will be pressure in terms of groundwater extraction. Dark blue areas indicate regions that are highly irrigated but have greater difficulty accessing deep groundwater. In A and B, groundwater availability is measured as the depth from the land surface to the point where groundwater begins (Verkaik *et al*., 2024). (C) Global mining pressure, illustrating potential hotspots where mining activities may reduce subterranean habitat availability. Mining intensity is calculated based on the percentage of each country's area occupied by mines (Tang & Werner, 2023) and the total extracted tonnage of target minerals (British Geological Survey, 2024). (D) Annual number of show cave visitors per country and associated income, estimated based on cave entrance fees (in dollars) (Chiarini *et al*., 2022).

Groundwater, the largest unfrozen continental reserve of fresh water globally (Gleeson *et al*., 2016; Ferguson *et al*., 2021), is a primary source of water for drinking, irrigation, and industrial use (Gleeson *et al*., 2020) (Fig. 4A,B). It is estimated that groundwater constitutes approximately 95% of the Earth's accessible liquid freshwater resources, including drinking water. Over a quarter of the global population relies on this resource, either partially or entirely (Taylor *et al*., 2013). Current human groundwater use is estimated to exceed the capacity of aquifers by about 3.5 times and groundwater decline is accelerating at a global scale (Jasechko *et al*., 2024). About 43% of irrigation water and 49% for domestic use is sourced from groundwater (Kuang *et al*., 2024), and this figure is likely to become even greater due to continuous population growth and increasing frequency of droughts and extreme events connected with climate change (Wu *et al*., 2020; Kuang *et al*., 2024).

However, there are large uncertainties in global estimations of the total volume of groundwater (Gleeson *et al*., 2016; Ferguson *et al*., 2021), its distribution (Gleeson *et al*., 2016), depth (Reinecke *et al*., 2024), recharge rates (Reinecke *et al*., 2021), and human extraction patterns (Loaiciga & Doh, 2024). Besides quantity, water quality is relevant; this depends primarily on geochemical processes and anthropogenic impacts but also, at least partially, on the presence of subterranean organisms (see Section IV).

### (2) Energy production

Subterranean ecosystems are increasingly used for heating, cooling, and direct energy production. Geothermal heat pump systems, which use heat from shallow underground sources, are the fastest‐growing segment of geothermal technology and one of the fastest‐growing renewable energy options. Other direct uses, such as heating buildings, bathing, swimming, industrial processes, farming (especially greenhouses), and fish farming, generally involve deep hydrothermal resources (Rybach, 2022). Deep geothermal energy plants produce hot water, directly used for heating purposes (e.g. *via* district heating networks) or turn the heat into electrical power. The global energy production *via* geothermal power plants of 95 TWh year^−1^ represents about 1% of the sustainable electricity generated annually. Hydropower, wind power, solar power, and power from biomass account for 59%, 19%, 13%, and 8%, respectively (Huttrer, 2020; Murdock *et al*., 2021; Rybach, 2022). However, among all renewables, geothermal power has the highest potential in the future, and an extraordinary high annual availability (60%) compared to other sources (Rybach, 2022).

For subterranean ecosystems, it is the use of shallow geothermal energy that is most relevant. In geology, the boundary between ‘shallow’ and ‘deep’ is typically set at a depth of 400–500 m, which corresponds, with only a few exceptions, to the deepest known occurrence of subterranean fauna (Fišer, Pipan & Culver, 2014). Most geothermal heat pump systems operate with relatively shallow closed‐loop borehole heat exchangers, often complemented by open, groundwater‐based systems. The shallow subsurface is warmer in winter and cooler in summer compared to the outside air. By using geothermal heat pump systems, this temperature difference can provide heating in winter and cooling in summer. However, it is important to note that extracting heat or cold from the subsurface can alter thermal conditions in ways that may be ecologically harmful. It can also induce temperature fluctuations resembling surface seasonality, although with much smaller temperature differences. Among these effects, warming is the main hazard for subterranean communities (Vaccarelli *et al*., 2023). Warming accelerates the metabolism of both microbes and fauna, leading to faster consumption of dissolved oxygen and potentially resulting in hypoxic or anoxic conditions. These oxygen‐depleted conditions can lead to the disappearance of fauna and are followed by a decline in water quality (Griebler *et al*., 2016). Thus, use of geothermal energy can conflict with the health of subterranean ecosystems, alongside other global drivers of subsurface warming such as climate change (Benz *et al*., 2024) and urbanisation (Bayer *et al*., 2016; Becher *et al*., 2022).

### (3) Food production

Groundwater is critical for global food security, supplying over 40% of the water used for irrigation and supporting approximately 13% of total food production (de Graaf *et al*., 2019) (Fig. 4B). Groundwater enables both large‐ and small‐scale farmers to enhance agricultural output, particularly in regions where rainfall is insufficient to meet crop water demands (Davis *et al*., 2017). Even though negative effects of irrigation can be mitigated (Carlson *et al*., 2025; Fišer *et al*., 2025), groundwater resources are increasingly overexploited, especially in major agricultural regions such as California's Central Valley, the High Plains Aquifer in the U.S. Midwest, the Middle East, the Indus and Ganges Basins, and the North China Plain (Famiglietti, 2014). Currently, India is the world's largest consumer of groundwater, supplying approximately 60% of its irrigation needs (Rodell, Velicogna & Famiglietti, 2009). Among internationally traded crops, rice is the most groundwater intensive, accounting for 29% of global usage, followed by wheat (12%), cotton (11%), maize (4%), and soybeans (3%). Citrus and sugar crops each also account for approximately 5% of groundwater use for irrigation (Dalin *et al*., 2017).

Beyond these agricultural trends, subterranean environments have been central to food production and foraging practices for millennia. Shepherds have historically used caves and caverns as shelters to protect livestock from harsh weather conditions (Hernández‐Marrero *et al*., 2016; Delhon, Martin & Thiébault, 2024). Additionally, caves were integral to traditional food preservation and production, particularly in the cheese and wine‐making industries, as well as mushroom cultivation, where their stable temperatures and humidity make them natural analogues to cellars (Pardo & Guerrero, 2006; Krishnan *et al*., 2019). As an example, the fungus *Penicillium roqueforti* was first discovered in limestone caves above Roquefort, France, where it accidentally transformed cheese into a flavourful delicacy, now renowned as Roquefort cheese.

Subterranean ecosystems also contribute to the service of food production by enabling aquaculture or as habitat for edible species. For example, anchialine pools have been used to keep fish for fresh consumption or even to cultivate fish bait used in traditional mackerel fisheries (Maly & Maly, 2003), as in the case of the red shrimp *Halocaridina rubra* (ʻōpaeʻula) in Hawaiʻi (Keliipuleole, 2022). The nests and eggs of cave swiftlets (*Collocalia linchi*) and Cory's shearwaters (*Calonectris diomedea*) are harvested for their nutritional (Yan *et al*., 2021) or traditional medical value (Rathi, Kumar & Vo, 2021). Oilbirds (*Steatornis caripensis*) are exploited in South America for their flesh and fat (oil), used for cooking and lighting (Brinkløv & Warrant, 2017). Bats are hunted as a meat source in Asia and Africa (Mickleburgh, Waylen & Racey, 2009). Depending on species and locations, bats are either considered a delicacy or an affordable source of protein during times of food scarcity (Jenkins & Racey, 2008; Kamins *et al*., 2011). However, such practices may threaten endangered species and their habitats (Tanalgo *et al*., 2023).

### (4) Raw materials

Rock, mineral, and materials extracted from subterranean ecosystems account for a major part of the global economy (Fig. 4C). The effects of mining, including rock or mineral extraction itself and all the infrastructure involved, potentially influences 50 million km^2^ of the Earth's surface (Sonter *et al*., 2020). In 2025, the global production of minerals is expected to reach 15 billion tons (Statista, 2025), with a value exceeding 7 trillion US$ in 2024 and constituting an important part of the national gross domestic product (GDP) in many countries (Reichl & Schatz, 2024).

Many of the mining areas coincide with protected, key biodiversity and wilderness areas. Hence, mining activities impact subterranean ecosystems, either directly (e.g. loss of habitat) or indirectly (aquifer contamination) (Mammola *et al*., 2019; Nanni *et al*., 2023). For example, iron ore production in Brazil accounts for approximately 1.6% of the country's GDP, generating around 31 billion US$ in 2022. With thousands of caves associated with iron ore landscapes, mining activities severely threaten these unique subterranean ecosystems, which are recognised for their significant diversity of cave‐restricted species (Ferreira, de Oliveira & Silva, 2018; Ferreira *et al*., 2022).

A special case of mining involves bat and bird guano, which can be locally abundant: millions of bats gathering in cave colonies can produce guano piles as high as 10 m (Cleary *et al*., 2022). Guano is widely used as a fertiliser due to its high nitrogen and phosphorus content (Sakoui *et al*., 2020), or as a source of chitin and chitosan for cosmetics, pharmaceutics, and textiles (Rinaudo, 2006; Kaya *et al*., 2014). Bat guano fertiliser typically costs US$ 2.50–24.00 per kg (Sakoui *et al*., 2020).

### (5) Biomolecular resources and emerging technologies

Subterranean ecosystems are a promising source of molecules and compounds with biotechnological applications (e.g. Cheeptham *et al*., 2013; Kosznik‐Kwaśnicka *et al*., 2022; Ciric & Šaraba, 2025).

Subterranean microbial biofilms often influence mineral precipitation and dissolution (Riquelme *et al*., 2015), particularly through polymeric substances that are produced and secreted by microbes (mediating microbial adhesion on surfaces) and may serve as nucleation sites for mineral precipitation, promoting the development of cave formations (Miller *et al*., 2012; Paar *et al*., 2016; Sauro *et al*., 2018). Secondary metabolites produced by microbes within those biofilms may have biotechnological and pharmaceutical applications, including use as enzymes, biosurfactants, or as antitumoral, immunosuppressive, and immunostimulatory agents (Sauro *et al*., 2018; Zada *et al*., 2021; Zhu *et al*., 2021; Ghezzi *et al*., 2022, 2024b; Pipite *et al*., 2022; Gatinho *et al*., 2024; Salazar‐Hamm *et al*., 2025). Some subterranean microorganisms with extracellular hydrolytic activity and antimicrobial compound production may be relevant against multidrug‐resistant pathogens (Cheeptham *et al*., 2013; Zada *et al*., 2021; Kosznik‐Kwaśnicka *et al*., 2022; Ghezzi *et al*., 2024b). For example, extracts of bacterial isolates from lava tubes of Lanzarote (Canary Islands) and orthoquartzite caves on Venezuelan tepuis showed antimicrobial activity against pathogenic strains of *Staphylococcus aureus*, *Escherichia coli*, *Pseudomonas aeruginosa*, and *Klebsiella pneumoniae* and exhibited antiproliferative activity against human breast cancer cells (Gatinho *et al*., 2024; Ghezzi *et al*., 2024b). While the therapeutic potential of metabolites produced by cave microorganisms has been extensively reported, to date the study of these molecules is still in its early stages (initial discovery and activity screening), and no clinical trial applications have been reported yet.

Beyond microbes, larger subterranean organisms have also been explored for their biomolecular potential. For example, many sessile invertebrates in marine caves (e.g. sponges, anthozoans, bryozoans, and tunicates) contain or secrete compounds with significant application potential (Uriz *et al*., 1991; Turon, Martí & Uriz, 2009; Audoin *et al*., 2013; Rotter *et al*., 2021; Suárez‐Moo *et al*., 2024). This biotechnological potential may also arise from more subtle interactions between microscopic and macroscopic organisms. For instance, animal excrement in caves, which often harbours pathogenic viruses, may stimulate microorganisms to produce antiviral substances (Gatinho *et al*., 2023).

Finally, the unique biological adaptations of several subterranean species hold promise for biomimicry, particularly in developing sensors, biomaterials, adhesives, and biologically inspired robotic movement (Hesselberg, 2023). In recent years, medical applications inspired by subterranean adaptations have also gained attention, ranging from potential treatments for diabetes (Riddle *et al*., 2018) and autism (Yoshizawa *et al*., 2018) to innovations in blindness research (Gore *et al*., 2018). Despite these possibilities, this potential remains largely untapped, with most studies still far from yielding concrete applications.

## IV. REGULATION & MAINTENANCE SERVICES

Regulation & Maintenance services provide the abiotic and biotic processes and environmental conditions that benefit living organisms, including humans (Haines‐Young & Potschin‐Young, 2018). Hence, these services offer stability, safety, and resilience to both ecosystems and human societies, and subterranean ecosystems contribute to as many as 82% of these.

### (1) Regulation of physico‐chemical conditions

Subterranean ecosystems are central to global water and (bio)geochemical cycles, including carbon, nitrogen, and other key elements (e.g. phosphorus, sulphur, and iron) (Griebler & Lueders, 2009; Edwards *et al*., 2012; Gleeson *et al*., 2020). Given their role in maintaining fresh water, sea water, and atmospheric balance, subterranean ecosystems are increasingly recognised as vital to global sustainability efforts. In particular, subterranean environments may be integral to Earth System governance frameworks such as the planetary boundaries, where groundwater has already been proposed as a key component (Gleeson *et al*., 2020). The planetary boundaries define a set of critical biogeophysical processes that collectively regulate the stability and resilience of the Earth System (Rockström *et al*., 2009; Steffen *et al*., 2015).

Hotspots for these biogeochemical processes are typically located along environmental gradients, redox interfaces, ecotones, and other transition zones in both terrestrial (e.g. subsurface–surface atmosphere, sediment/rock–atmosphere interfaces) and aquatic settings (e.g. land–sea, sediment–water, and water–atmosphere interfaces) (McClain *et al*., 2003). These environmental gradients span micro (< mm) to regional scales (> km), and their role in regulating chemical fluxes and ecosystem functioning is often disproportionately large relative to their size (Schmidt, Cuthbert & Schwientek, 2017). Some of these processes are also mediated within the so‐called ‘deep biosphere’ following the recognition that bacteria and archaea occur kilometres deep in the Earth's crust (Pedersen, 2000). Yet, major gaps remain in our understanding of their extent, function, and role in global biogeochemical cycling (Edwards *et al*., 2012).

Biogeochemical processes associated with subterranean ecosystems primarily regulate the chemical conditions of freshwater and marine habitats. Natural and anthropogenic inputs of nutrients and organic matter from the surface into the groundwater increase dissolved organic carbon (DOC) and nitrate concentrations, both important indicators of water quality, that are then attenuated through microbial activity (Griebler & Avramov, 2015). For instance, redox‐driven microbial processes under aerobic or anaerobic conditions (e.g. denitrification and iron reduction) can consume substantial amounts of nitrate and reduce or transform DOC as groundwater migrates through subterranean freshwater environments (Chapelle, 2000) or discharges into the sea (Santos *et al*., 2021). Marine caves and cavities in tropical regions are also areas of heterotrophic DOC consumption (de Goeij *et al*., 2008), which depletes dissolved oxygen (Young *et al*., 2018).

Fresh groundwater discharge only accounts for a minor portion (~0.6%) of the total freshwater input to the world's oceans (Luijendijk, Gleeson & Moosdorf, 2020), but it can be critical locally for coastal ecosystem functioning due to its solute and nutrient loads (Slomp & Van Cappellen, 2004). At the land–sea interface, the region of a coastal aquifer where sea water and groundwater mix is the subterranean estuary, which often hosts anchialine ecosystems (Bishop *et al*., 2015). The subterranean estuary is an important biogeochemical reaction zone that modulates nutrient and carbon fluxes from rocky, sandy, and muddy coastlines to marine ecosystems and fisheries (Moore, 1999; Santos *et al*., 2021). For example, microbial activity reduces nitrate and methane concentrations in groundwater discharged from sandy coasts (Santos *et al*., 2008; Schutte *et al*., 2016), and methane and DOC in groundwater discharging from karstic coastlines (Brankovits *et al*., 2017, 2018). Moreover, sinkholes along karst coastlines are hotspots for carbon burial (Adame *et al*., 2021), highlighting their potential for inclusion in blue carbon stocks. Given that approximately 40% of the world's population lives within 100 km of the coast (United Nations Environment Programme, 2021), understanding these dynamics is of growing global importance.

Furthermore, subterranean microbes and metazoans can impact and modify the subterranean environment itself, for example by generating habitat space. As noted in Section III.5, microbial activity and their metabolic products can induce rock dissolution or mineral precipitation. For instance, nitric acid can be produced in caves by bacteria that oxidise ammonia derived from bat and bird guano. This nitric acid dissolves the limestone bedrock, leading to the formation of distinctive dissolution features (such as megascallops and streamlined fluting), which in turn modify cave morphology (Farrant *et al*., 2025). These effects can be significant over long timescales (>22,000 years); indeed, bats in tropical caves have recently been described as ‘ecosystem engineers’ (Pilò *et al*., 2023).

Beyond biogeochemical cycles, subterranean ecosystems regulate key physical conditions in the environment. Hydrogeological conditions in aquifers control land subsidence, a phenomenon mainly driven by excessive groundwater extraction and aquifer compaction. Globally, land subsidence leads to the loss of aquifer storage (~17 km^3^/year) and affects mainly cropland and urban areas (73%) (Hasan *et al*. 2023). Consequences include damage to infrastructure and increased flood hazards, with substantial economic and human impacts (Erkens *et al*., 2015; Connor & Miletto, 2022). Groundwater also provides essential baseflow to rivers, particularly during dry seasons; globally, baseflow is estimated to account for a mean ± SD of 59 ± 7% of river flow (Xie *et al*., 2024). Groundwater also supports services provided by groundwater‐fed vegetation, such as water storage, purification, and flood control. In turn, groundwater‐fed vegetation controls erosion rates (Lowry & Loheide, 2010), regulates the overall hydrological cycle and water flow, and contributes to flood control and coastal protection. The value of groundwater‐fed vegetation in flood control has been estimated at about €16 billion in the EU alone (Vallecillo *et al*., 2020).

### (2) Regulation of biological conditions

Subterranean ecosystems support surface vegetation (Glanville *et al*., 2023; Saccò *et al*., 2024) and marine habitats (Moore, 2010; Santos *et al*., 2021). Approximately 37% of the world's vegetation depends on groundwater to some extent (Barbeta & Peñuelas, 2017; Evaristo & McDonnell, 2017). The quality and availability of groundwater influence the distribution, diversity, functioning, and resilience of these plant communities (Glanville *et al*., 2023). This dependency is particularly pronounced in drought‐prone regions, where threshold levels of groundwater availability serve as indicators of potential drought refugia (Rohde *et al*., 2024). Groundwater discharged into the marine environment delivers nutrients and affects water quality in estuaries, coral reefs, lagoons, mangroves, and saltmarshes (Moosdorf *et al*., 2021; Santos *et al*., 2021).

Subterranean ecosystems also act as temporary, daily, or seasonal habitats for many surface animals and plants, all of which are integral to interconnected subterranean–surface food webs (Mammola, 2019). Surface vertebrates shelter or nest in cave entrances (Baker, 2015; Tuniyev, Koval & Vargovitsh, 2021; dos Santos *et al*., 2022; Hernández‐Lozano *et al*., 2024), while bats mate near entrances, but breed and hibernate in deeper sections (Furey & Racey, 2016). Different vertebrates and invertebrates move in and out of terrestrial caves, often guided by circadian rhythms or seasonal cues (Novak, Thirion & Janžekovič, 2010; Mammola & Isaia, 2018; Moog, Christian & Eis, 2021; Tuniyev *et al*., 2021). Aquatic insects, crustaceans, and fish seek refuge in the hyporheic zone of rivers during droughts (Amoros & Mathieu, 1976; Rouch & Danielopol, 1987; Palmer, Bely, & Berg, 1992; Land & Peters, 2023). Groundwater inputs also heavily influence freshwater fish behaviour, migration, spawning, and distribution (Land & Peters, 2023). Similarly, marine caves host diverse sessile invertebrates (e.g. sponges, corals, bryozoans and brachiopods), fishes, and crustaceans, including many economically and ecologically valuable species such as the precious red coral *Corallium rubrum* (Gerovasileiou & Bianchi, 2021). As climate becomes more unpredictable, these subterranean refugia are expected to grow in importance because of their thermal stability (Vaccarelli *et al*., 2023).

Arguably, cave‐dwelling bats represent the best‐studied example of biological regulation by subterranean ecosystems. Bats provide critical pollination and seed dispersal services for economically important plants, including figs, durian, mango, and agave (Medellin *et al*., 2017; Ramírez‐Fráncel *et al*., 2022). For instance, the pollination services of *Eonycteris spelaea* to durian farmers in Sulawesi, Indonesia, were valued at US$117 per hectare during each fruiting season (Sheherazade & Tsang, 2019). Another notable example is the mutualistic relationship between bats and agave. The pollination of agave relies on bats, particularly the cave‐dwelling *Leptonycteris nivalis*, which, in turn, depend on agave during their seasonal migrations (Trejo‐Salazar *et al*., 2023). Agave holds cultural and economic significance in Mexico as a source of food, spirits (tequila and mezcal), and fibre.

Insectivorous bats are also key biological controllers due to their hunting efficiency. For example, the cave‐dwelling species *Pteronotus gymnonotus* and *P. personatus* consume 5–28% of their body mass in insects each night (Pimentel *et al*., 2022). At least 81 species of insectivorous bats, including several obligate or facultative cave‐dwellers, prey on over 760 species of insect pests that affect economically important crops such as corn, coffee, cotton, rice, apples, macadamia nuts, cocoa, and grapes (Tuneu‐Corral *et al*., 2023). Some of these species form massive colonies. For example, Mexican free‐tailed bats (*Tadarida brasiliensis*) can form colonies of millions of individuals. During the summer, when bat populations peak in Bracken Cave, Texas, they can remove approximately 100 tons of insects per night, with the annual value of this pest suppression estimated at US$3.42 million (Medellin *et al*., 2017). The economic importance of insectivorous bats in northern America has been estimated to be as high as exceeding US$3.7 billion per year (Boyles *et al*., 2011).

### (3) Mitigation of pollutants

The Chemical Abstracts Service lists >200 million organic and inorganic synthesised compounds, with 20,000–30,000 new entries added daily (Chemical Abstracts Service, 2025). Many of these chemicals, especially those produced in large volumes, are released into the environment and eventually make their way underground, either passively (e.g. through percolating water) or intentionally (historically, shallow aquifers and caves were often used as waste disposal sites) (Lapworth *et al*., 2022; Misstear *et al*., 2023).

Against this backdrop, a critical service is self‐purification. This refers to the removal or immobilisation of pollutants through natural processes, as well as the regulation of substances of non‐anthropogenic origin that can become problematic for the environment when present in excessive quantities or concentrations (e.g. detritus, nutrients) (Griebler *et al*., 2019). Subterranean microorganisms are key actors in this process, transforming harmful substances into more stable or less toxic forms. For example, bacteria such as *Alcaligenes*, *Acinetobacter*, and *Pseudomonas* can immobilise heavy metals or dissolve phosphate minerals, aiding in the removal of contaminants (Putilina & Yuganova, 2022). As for organic pollutants, microbes can degrade or mineralise compounds like petroleum hydrocarbons and halogenated solvents, particularly in point‐source contamination scenarios (Das & Chandran, 2011). For instance, in microcosm experiments mimicking groundwater flow through sand in aerobic and anaerobic conditions, *Pseudomonas putida* aerobically degraded 35% of toluene within 15.5 h over 78 cm, while *Aromatoleum aromaticum* achieved 98% removal under nitrate‐reducing conditions (Bauer *et al*., 2008). However, these processes are often slow, as microbial activity in subsurface environments is limited, and groundwater contamination can persist for years (Tatti *et al*., 2019; Li *et al*., 2021). For example, nitrates persist in groundwater for decades unless hypoxic or anoxic conditions and an appropriate electron donor (e.g. organic matter, pyrite) are present (Basu *et al*., 2022).

Self‐purification processes may be stimulated *via* amendment of electron acceptors (e.g. dissolved oxygen), electron donors (e.g. molasses), and bacterial strains (termed bioaugmentation) (Logeshwaran *et al*., 2018). Managed aquifer recharge systems can effectively remove contaminants (Laws *et al*., 2011), including pharmaceuticals and antibiotics, through degradation processes that depend on the aquifer's redox state and temperature (Burke, Duennbier & Massmann, 2013). However, biotransformation processes can sometimes produce byproducts that are recalcitrant to further degradation or more toxic than their parent compounds, highlighting the complexity of chemical regulation in groundwater systems (Postigo & Barceló, 2015).

Beyond microorganisms, larger subterranean fauna may also contribute to water purification through bioturbation of sediments and filtration (Boulton *et al*., 2008; Hose & Stumpp, 2019). Based on consumption rates and rough density estimates of the isopod *Phreatoicus typicus* in New Zealand, it has been estimated that a population of 100 individuals can process approximately 7–28 tonnes of sediment per hectare annually and assimilate 120–650 g of organic carbon per hectare annually (Boulton *et al*., 2008). Similarly, laboratory estimates suggest that groundwater‐obligate asellid crustaceans can process 1.1–16.4% of the available organic carbon in their environment each day, indicating that, despite inhabiting energy‐poor habitats and having lower processing rates than surface species, they still provide significant carbon‐processing services comparable to their surface‐water counterparts (Mermillod‐Blondin *et al*., 2025). Synergistic effects with microorganisms appear to be particularly important in this context. Amphipods, isopods, and other invertebrates bioturbate and aerate sediments, creating favourable conditions for microbial communities to degrade contaminants (Malard & Hervant, 1999; Boulton *et al*., 2008; Hose & Stumpp, 2019). For example, the isopod *Coecidotaea tridentata* enhances both planktonic and sedimentary bacterial abundance and activity through the excretion of nitrogen, which promotes microbial growth, the disturbance of sediments, and the direct consumption of bacteria (Edler & Dodds, 1996). Indeed, a recent modelling study suggested that the absence of groundwater‐obligate fauna can drastically reduce microbial activity and carbon degradation, leading to up to 660 times more unrecycled organic carbon compared to systems where fauna are present (Schmidt, Rütz & Marxsen, 2025).

### (4) Potential for climate change mitigation

Subterranean ecosystems, particularly karst environments and caves, play a surprisingly important yet understated role in locally regulating atmospheric composition. Silicate weathering in the subsurface is strongly controlled by groundwater dynamics. These processes, among others, generate alkalinity in groundwater that is exported to rivers and oceans, acting as a major long‐term sink for atmospheric CO_2_ and stabilising Earth's climate over geological timescales (Zhang & Plavansky, 2019; Brantley *et al*., 2023). Middelburg, Soetaert & Hagens (2020) suggested the global export of alkalinity to the oceans is about 1.2 Tmol/year, providing a key link between terrestrial weathering and the marine carbon cycle.

Moreover, microbially mediated formation of speleothems, such as moonmilks, sequesters and stores CO_2_ (Martin‐Pozas *et al*., 2022; Ghezzi *et al*., 2024a). Furthermore, aerobic caves act as net sinks for atmospheric methane (CH_4_), actively consuming this greenhouse gas through microbial oxidation mediated by methanotrophic bacteria (Waring *et al*., 2017; Webster *et al*., 2018; Ojeda *et al*., 2019) or through other processes (Fernandez‐Cortes *et al*., 2015). Within flooded caves of a karst subterranean estuary, it is estimated that ~1.4 tons of methane was consumed during 6 months across a ~100 km^2^ catchment region in the Yucatán Peninsula (Brankovits *et al*., 2018). It is unlikely that this magnitude of methane removal would affect global greenhouse gas budgets, but it quantifies the contribution of a critical energy source for an anchialine food web (Brankovits *et al*., 2017).

Beyond gas fluxes, subterranean ecosystems influence microclimatic conditions. Their ability to buffer temperature and maintain high humidity levels creates stable environments that interact with aboveground climates, especially in regions with extensive karst topography (Caldwell *et al*., 2020; Goldscheider *et al*., 2020). In terrestrial systems, this kind of regulation is often aided by bryophyte cushions (mosses and liverworts) developing in the entrance zone of caves, which function as living sponges, intercepting rainfall, fog, and dew and retaining water volumes several times their dry mass. By slowly releasing this stored moisture into the substrate and underlying fissures, they buffer hydrological extremes at the subterranean–surface interface, sustain high local humidity for microbial and faunal communities, and contribute measurably to the water‐storage service of groundwater‐dependent ecosystems (Cedrés‐Perdomo *et al*., 2024). In aquatic and marine settings, flooded caves and other subterranean environments have an important role in heat transfer through groundwater transport. Aquifers in rocky coastlines, such as karstic and volcanic platforms, have distinct properties, because the fissures and conduits enhance hydraulic transport and exchange of material with the sea through diffuse processes or submarine springs (Fleury, Bakalowicz & De Marsily, 2007; Moore, 2010; Moosdorf & Oehler, 2017). Tidal‐driven oscillation of fresh groundwater discharge has been shown to transport heat to the sea from a volcanic platform (Taniguchi, Ishitobi & Shimada, 2006). On the contrary, tropical carbonate platforms may cool the nearby sea through fresh groundwater discharge while facilitating the marine‐derived saline water to import heat from the coast to inland (Beddows *et al*., 2007).

Beyond caves, groundwater‐dependent ecosystems such as groundwater‐fed wetlands, fens, riparian forests, and woodlands facilitate atmospheric CO₂ uptake through photosynthesis, root respiration, bicarbonate formation in soil, and the subsequent storage of carbon in groundwater or its precipitation as calcium carbonate (Singh *et al*., 2023). Vegetation supported by groundwater, such as the redwood forests of northern California, grows more robustly and for longer periods compared to vegetation without groundwater access, sequestering significantly more carbon (Howard *et al*., 2023). Notably, areas with groundwater‐dependent ecosystems store approximately 790 million tons of CO₂ – nearly double California's annual emissions (Howard *et al*., 2023). However, these benefits can be counterbalanced by the dewatering of groundwater‐dependent ecosystems. For example, estimates suggest that wetlands could emit ∼408 gigatons of CO₂ between 2021 and 2100 if degraded or drained (Zou *et al*., 2022).

## V. CULTURAL SERVICES

Cultural ecosystem services are the non‐material benefits people derive from ecosystems, contributing to cultural identity, spirituality, scientific endeavours, and quality of life (Haines‐Young & Potschin‐Young, 2018). Subterranean ecosystems contribute to all of these services.

### (1) Tourism and recreation

Terrestrial and marine caves are among the most frequently visited geo‐ and ecotourism attractions worldwide. A recent synthesis identified 1223 show caves across 95 countries, involving an estimated 79 million visitors in 2019 (Chiarini *et al*., 2022). This generates around €800 million in entrance fees, with an even greater economic impact when considering related tourist activities such as souvenir shops, restaurants, bars, and local transport (Fig. 4D). Inevitably, this level of tourism comes with impacts, including structural damage to caves, alterations to local climatic conditions, the introduction of external organic matter and non‐native fungi, bacteria, and animals, and the growth of photosynthetic organisms due to artificial lighting (Piano *et al*., 2024). Since several show caves also host a high number of species (e.g. Deharveng *et al*., 2024), this raises the question of whether the management of show caves has a positive or negative impact on subterranean biodiversity.

Furthermore, geothermal phenomena linked to subterranean ecosystems, such as boiling lakes, mud ponds, and geysers, serve as striking natural attractions, drawing visitors to destinations that blend wonder with recreation. Some of these features also fuel the wellness sector. Thermal springs, long used by humans – and other apes (Matsuzawa, 2018) – for health and wellness, are increasingly being transformed into modern hot spring resorts and water parks. Similarly, speleotherapy, particularly speleoclimatotherapy and radon therapy, offers drug‐free therapeutic benefits. For example, the unique microclimate of salt caves and mines, often characterised by fine aerosols of NaCl, K^+^, and Mg^2+^, high humidity, low radiation, light air ions, hypoallergenic air, and a stable temperature, can alleviate different respiratory syndromes (Munteanu, 2017).

Terrestrial and aquatic caves are popular recreational sites for activities such as caving, snorkelling, scuba diving, and boat tours (Gunn, 2004; Wilson, 2019). These activities range from spontaneous experiences lasting a few hours, undertaken solo or in groups, to more structured expeditions and cave trips that require advanced speleological knowledge and skills. Often this kind of tourism brings visitors to caves that would be closed to humans otherwise, which may cause local impact to the ecosystems but also enhance scientific knowledge by citizens, amateur scientists, and speleologists.

Finally, subterranean‐related ecotourism offers opportunities for wildlife enthusiasts to observe animals in their natural habitats. For instance, bat‐watching is increasingly popular worldwide (Kunz *et al*., 2011). The nightly emergence of millions of Mexican free‐tailed bats from caves in the southwestern USA is estimated to attract over 240,000 visitors each year, conservatively valued at $6.5 million annually (Bagstad & Wiederholt, 2013). Such activities support local economies and provide unique educational experiences for the public, raising awareness about the ecological significance of subterranean fauna.

### (2) Aesthetic and artistic value

Subterranean landscapes inspire and support a range of artistic expressions (Gleeson, 2024; Mammola *et al*., 2025). For instance, artistic practices have explored groundwater as a theme through creative expressions of its sensory qualities – tastes, smells, sounds, textures, and movements – as well as its landscapes, cultural significance, and community connections (Gleeson, 2024). Contemporary abstract art frequently draws from the textures and patterns of speleothems, as seen in the cave‐inspired works of artist Ana Teresa Barboza. Literature has frequently embraced subterranean themes, such as Jules Verne's *Journey to the Centre of the Earth* and Haruki Murakami's *Hard‐Boiled Wonderland* and the *End of the World*. Music, too, draws inspiration from subterranean acoustics, with composers like John Luther Adams creating pieces that echo the resonant and mysterious qualities of caves. Architecture has similarly demonstrated how caves and sinkholes can be reimagined into cultural and artistic venues, with spaces like *Los Jameos del Agua* in Lanzarote, shaped by César Manrique and Jesús Soto. These are just a few examples among many (Gleeson, 2024; Mammola *et al*., 2025).

It has been argued that subterranean‐related art may improve scientific communication and support the conservation of subterranean ecosystems (Danielopol, 1998; Gleeson, 2024; Mammola *et al*., 2025). For example, projects such as the virtual reconstructions of cave art by the Chauvet Cave team not only preserve these fragile environments but also educate the public about their ecological and historical significance. Likewise, the Cenoteando initiative (https://cenoteando.mx/) in Mexico has developed several educational materials that combine scientific accuracy with artistic expression to promote environmental awareness and proper stewardship of cenotes, enabling a sustainable interaction with these fragile environments. Similarly, artworks and photography that highlight the fragility of subterranean ecosystems, such as those by environmental artists like Agnes Denes and Martin Broen, can galvanise support and financial backing for conservation campaigns. Lastly, there is a practical significance to exploring aesthetics of subterranean features. For example, groundwater aesthetics – taste, odour, colour, and clarity – is essential in shaping cultural perceptions and public trust in water supplies (Burlingame *et al*., 2024).

### (3) Scientific research

Terrestrial caves have long been regarded as model systems for scientific research across various fields (Poulson & White, 1969; Martinez & Mammola, 2020; Mammola *et al*., 2020). The convergent adaptations of subterranean organisms make subterranean ecosystems a rich subject for evolutionary research, with a lineage of studies tracing back to Charles Darwin (Juan *et al*., 2010). Several cave‐adapted species, such as cavefish and crustaceans, serve as established model organisms for evolutionary studies and beyond (Mammola *et al*., 2021). Furthermore, due to their climatic stability, low biological diversity, simple habitat structure, and often isolated nature, caves allow researchers to minimise many confounding factors that typically complicate ecological studies in surface environments (Mammola, 2019). Similarly, marine caves in the littoral zone have been described as ‘deep‐sea mesocosms’, providing direct human access to deep‐sea‐like conditions (Harmelin & Vacelet, 1997).

Importantly, this expanding research agenda builds upon the observations made by individuals who regularly explore subterranean environments, often driven by personal passion and a deep appreciation for nature. Speleological and cave diving clubs are typically composed of highly experienced, non‐scientific explorers who possess the technical expertise necessary to access and map these underground spaces. Scientific research is also increasingly supported by dedicated subterranean research facilities, such as the Moulis Experimental Ecology Station in France and the Boulby Underground Laboratory in the UK, which provide controlled environments for ecological and evolutionary experiments (Mammola *et al*., 2021). Other underground laboratories, including Gran Sasso (Italy) and SNOLAB (Canada), further highlight the broader scientific value of caves, extending beyond biology to fields such as astroparticle physics.

Beyond biological research, caves play a crucial role in archaeology and palaeontology by safeguarding fossils, sediments, prehistoric artifacts, and even recently extinct species, such as certain birds known only from cave deposits in Macaronesia (Rando *et al*., 2013, 2017), as well as numerous human remains discovered in caves around the world (e.g. Chatters *et al*., 2014; Berger *et al*., 2015). Stalagmites are archives for paleoclimate research, offering high‐resolution records of past climatic fluctuations through isotopic and geochemical analyses (Fairchild & Baker, 2012), while sediment deposits within cave systems record paleoenvironmental history, such as changes in sea level (van Hengstum *et al*., 2010, 2011, 2019). All these natural archives provide clues into past ecosystems useful for reconstructing paleoenvironments and their biodiversity, yielding important implications for establishing baseline references for conservation and restoration efforts (Hughes *et al*., 2023). For example, the analysis of speleothems has provided evidence of past environmental changes and the anthropogenic impacts that contributed to the well‐documented ecocide on Easter Island (Miller *et al*., 2016). Similarly, speleothems from lava tubes in the Galapagos Islands have revealed biomarkers of surface vegetation changes and human‐induced pollution, emphasising the need for robust conservation policies to mitigate the impact of anthropogenic activities (Miller *et al*., 2022).

The inspirational value of caves may even extend beyond Earth (Sauro *et al*., 2021, 2023; Titus *et al*., 2021). The detection of volcanic caves on Mars and their protective properties against surface radiation, extreme temperatures, and atmospheric variability, have led researchers to explore caves on Earth from planetary science and astrobiological perspectives. A rich research agenda is forming, showing that these subterranean environments could serve as analogues for space exploration and planetary research (Sauro *et al*., 2021; Wynne *et al*., 2022), and offer insights into the possibility of extraterrestrial life (Northup *et al*., 2011; Popa *et al*., 2011). Specifically, microbial metabolism and mineral interactions in caves and lava tubes on Earth generate a variety of biosignatures (Westall *et al*., 2015; Wynne *et al*., 2022; Palma *et al*., 2024a,b), which provide reference models for potentially detecting extraterrestrial microbial life (Macalady Jennifer *et al*., 2006). Moreover, deep caves offer opportunities for training for astronauts (e.g. the programme by the European Space Agency), allowing them to practice behaviour and tasks in harsh environments that resemble conditions in space.

### (4) Education

Subterranean ecosystems offer vast educational potential, especially for fostering scientific literacy and environmental awareness. Every cave provides visitors with an unforgettable experience, combining natural beauty with rich site‐specific educational opportunities. Cave interpretation centres, guided tours, and interactive activities can help students and visitors appreciate the uniqueness of cave ecosystems and the importance of their conservation. Similarly, groundwater‐fed springs enhance the natural beauty of their surroundings and serve as ideal settings for educational school trips. These sites allow students and teachers to observe firsthand the interactions between groundwater systems, biodiversity, and human activities (Reinfried *et al*., 2015). Activities such as water‐quality testing, species identification, and habitat mapping can transform these visits into living laboratories, offering hands‐on learning experiences that reinforce classroom lessons.

This interplay between natural and cultural elements creates opportunities for educational projects that explore connections across disciplines such as biology, earth sciences, history, and even art. For example, studying speleothems can teach students about geological processes, offering a concrete visual representation of time accumulation, while analysing the unique adaptations of cave‐dwelling organisms can illustrate fundamental evolutionary principles. Importantly, these educational activities can be reinforced through citizen science initiatives. A recent citizen science project collected biological samples from over 300 municipal groundwater sites across Switzerland. This initiative bridged educational objectives with research goals, leading to the discovery of new species (Alther *et al*., 2021) and enabling the mapping of macroecological patterns at unprecedented resolution (Schneider, Knüsel & Altermatt, 2023; Knüsel *et al*., 2024b; Knüsel, Alther & Altermatt, 2024a).

### (5) Cultural heritage and identity

Subterranean ecosystems often shape traditions, customs, and identities, influencing both positive and negative cultural narratives. Historically, caves were often perceived as liminal spaces, i.e. thresholds between the world of the living and the underworld. In European folklore, they often symbolise fear of the unknown and are believed to be entrances to Hell or lairs for dragons, trolls, and other sinister beings. This is illustrated in 17th‐century engravings published in a monograph on the Duchy of Carniola by J. V. Valvasor, a Slovenian scientist, which depicts the beliefs of local inhabitants at the time (Crane & Fletcher, 2015). Yet, caves have also held positive associations, for example by serving as places of refuge (Bertini, 2010). Quintessential examples are underground cities in the Mediterranean region, such as Matera (Italy), Bulla Regia (Tunisia), and Cappadocia (Turkey), with tunnels, living quarters, and even chapels carved into the rock. Similarly, Coober Pedy, South Australia, is renowned for its man‐made ‘dugouts’, subterranean residences bored into the hillsides of the desert. Beyond human‐accessible cavities, features such as springs, anchialine pools, and oases played vital roles in community life, fostering social interaction and cohesion.

Specific organisms, such as bats, are often protagonists of these cultural narratives (Sieradzki & Mikkola, 2022). In some traditions, bats are feared as harbingers of darkness and death, a view perpetuated by Gothic literature and popular media. However, bats are also revered as symbols of luck, fertility, or protection. For example, in Chinese culture, bats are associated with happiness and prosperity, as the word for bat (*fu*) sounds like the word for good fortune. In the Americas, indigenous communities such as the Maya often incorporate bats into their mythology, viewing them as powerful guardians of the underworld.

Slovenia offers a prime example of how, even today, subterranean landscapes and their fauna can be deeply intertwined with national identity. The country is home to the renowned Postojna Cave, a UNESCO‐listed site that has become a source of national pride (Zagmajster, Polak & Fišer, 2021). This is the cave where the first scientific descriptions of exclusive cave‐dwelling animals originated, beginning with the beetle *Leptodirus hochenwartii*, which marks the start of speleobiological research in 1832 (Schmidt, 1832). Slovenia is also the land where the discovery and scientific description of the olm (*Proteus anguinus*) took place. This species is a blind, pale groundwater salamander that has achieved iconic status, celebrated across various facets of Slovenian culture, from beer labels and public street art to the textile industry and contemporary art projects.

### (6) Spiritual and religious significance

Caves, anchialine pools, subterranean rivers, springs, and cenotes were often regarded as sacred or spiritually significant (Moyes, 2012; Ray, 2020). For example, the caves of Crete were religious sites for the ancient Minoans, while Zeus was believed to have been born in a cave. In Greek mythology, the river Styx delineated Hades, the underworld (the prefix ‘stygo‐’ is still used today for ‘stygobionts’, a technical term referring to groundwater‐dwelling organisms). Similarly, the cenotes of the Yucatán Peninsula were viewed by the Maya as both gateways to Xibalba, the underworld, and essential sources of life‐giving water (Munro & de Lourdes Melo, 2011; Melo Zurita, 2019). Likewise, many anchialine pools in Hawai‘i are revered as *wahi pana* (celebrated places), or strictly reserved for various uses, including royal baths, rituals, and ceremonies (Gibson *et al*., 2022).

Countless rock‐cut churches and monasteries worldwide further highlight the spiritual dimensions of subterranean sites (Bertini, 2010). Likewise, groundwater provides spiritual and religious services through sacred water sites, often linked to natural features such as trees, stones, caves, and hills. These places offer a sensory connection to spiritual practices, with holy wells and springs frequently serving as focal points for rituals and supernatural engagement. While not all water sources are considered sacred, many cultures believe in offering gifts to water spirits to sustain their blessings. Springs emerging from caves hold particular significance, often seen as miraculously pure and ritually powerful, with evidence of reverence spanning from prehistoric times to contemporary cultures worldwide (Ray, 2020). In Australia, many Aboriginal nations consider groundwater sites fundamental to their Dreamtime creation stories, in which the Rainbow Serpent is believed to have shaped landforms, springs, and river upwelling zones. Many sacred sites associated with fertility, teachings of lore, and cultural customs are linked to groundwater, holding immeasurable value for these communities (Moggridge, 2020).

## VI. THE ECONOMIC DIMENSION OF SUBTERRANEAN ECOSYSTEM SERVICES

Valuation of services provided by subterranean ecosystems is still in its early stages. A recent review of over 1300 studies, yielding more than 9400 monetary value estimates, found that subterranean ecosystems accounted for only 0.08% of the sample (Brander *et al*., 2024). Similarly, Kadykalo *et al*. (2021) reported negligible research effort towards subterranean ecosystems when analysing the correlation between ecological and economic assessments of 15 regulating services across 32 ecosystem types. While ecological roles such as nutrient cycling, soil formation, and groundwater provision are well documented, their economic valuation remains limited, with groundwater being the most studied (Kadykalo *et al*., 2021).

In subterranean ecosystems, most valuation efforts focus on provisioning services, particularly groundwater. Methods include market prices, replacement costs, and production functions that measure the marginal impact of water on economic outputs like agricultural crops (Aziz *et al*., 2023). However, market prices often fail to capture the full social value of groundwater due to distortions like subsidies, requiring adjustments to reflect true economic value (Singh *et al*., 2017). Replacement cost methods, which estimate the expenses needed to restore lost services, offer an alternative approach (Carrera‐Hernández & Gaskin, 2009).

Regulating services, although frequently reported for subterranean ecosystems, are rarely valued economically. For example, studies on erosion control, flood protection, and water quality regulation typically focus on surface ecosystems rather than subterranean ones (Patault *et al*., 2021; Lundin‐Frisk *et al*., 2024). Similarly, cultural services like geo‐ and ecotourism are gaining attention, with examples including the recreational value of mining heritage and willingness‐to‐pay estimates for geo‐guided tours (Cheung, Fok & Fang, 2014; Pérez‐Álvarez *et al*., 2016; Kubalíková, 2020; Khalaf, 2024).

## VII. SUBTERRANEAN ECOSYSTEM DISSERVICES

Alongside their many positive contributions, ecosystems can also have effects that are perceived as harmful, unpleasant, or unwanted: termed ‘ecosystem disservices’ (Blanco *et al*., 2019). While research on subterranean ecosystem disservices is virtually non‐existent and beyond the scope of this assessment, it is important briefly to mention the potential human health and infrastructural risks associated with these environments. For instance, subterranean ecosystems can serve as reservoirs of pathogens and facilitate disease transmission. They harbour harmful microbes, fungi, and viruses, which may exist freely or be associated with specific organisms. Cave‐roosting bats, in particular, are significant vectors of pathogens, including *Histoplasma* fungi found in bat guano, which can cause histoplasmosis in humans (Gugnani & Denning, 2023). Additionally, subterranean environments can accumulate potentially toxic gases such as carbon dioxide, methane, hydrogen sulfide, and radon. These gases pose risks of asphyxiation or poisoning, while radon may increase lung cancer risk for frequent visitors.

At the same time, the public's fascination with the underworld has often led to unfortunate accidents, particularly when individuals engage in caving or cave diving without adequate training or equipment, as in the famous Thailand cave rescue (Beech, Paddock & Suhartono, 2018) or the harrowing account of Sheck Exley in the Túnel de la Atlántida (Exley, 1994). Subterranean ecosystems can also evoke some of the most common human phobias, as ranked by Correia & Mammola (2024). These environments are often dark (nyctophobia), enclosed (claustrophobia), contain deep pits or abysses (acrophobia/vertigo), and host fear‐inducing organisms such as spiders (arachnophobia) and bats (chiroptophobia), potentially causing psychological distress in visitors.

Beyond direct health risks, subterranean environments also pose threats to human infrastructure. Natural underground erosion, combined with human activities such as mining and groundwater extraction, can lead to cave collapses and sinkholes, damaging buildings and roads.

This discussion of disservices is far from exhaustive. Yet, it serves as a placeholder for further research in this area. Indeed, studying ecosystem disservices has been proposed as a way to balance the benefits and drawbacks of nature better, ultimately leading to a more objective evaluation of its net impact on human well‐being (Schaubroeck, 2017).

## VIII. OUTLOOK: COMMUNICATING THE VALUE OF SUBTERRANEAN ECOSYSTEMS

Although still emerging, research on subterranean ecosystem services is likely to expand rapidly (Canedoli *et al*., 2022). We now have reasonable estimates of the global distribution and volume of certain types of subterranean ecosystems (Gleeson *et al*., 2016; Ferguson *et al*., 2021), a growing understanding of subterranean biodiversity patterns (Zagmajster *et al*., 2023; Martínez *et al*., 2024), and insights into the proportion of these ecosystems and their biodiversity that is protected (Sánchez‐Fernández *et al*., 2021; Mammola *et al*., 2022, 2024). Increasingly available open data (Huggins *et al*., 2025) and emerging technologies – from‐omics tools (Pérez‐Moreno, Iliffe & Bracken‐Grissom, 2016; Cappelletti *et al*., 2025) and environmental DNA (Saccò *et al*., 2022) to terrestrial laser scanning (Idrees & Pradhan, 2016) and computer simulations (Mammola *et al*., 2021) – enable us to map and quantify subterranean ecosystems at unprecedented resolutions. Simultaneously, state‐of‐the‐art economic theory provides a set of approaches to quantify the socio‐economic relevance of these services at meaningful scales. If harnessed effectively, these tools could bridge critical knowledge gaps in subterranean ecosystem services research.

Notwithstanding these advances, the importance of subterranean ecological processes to support surface ecosystems and human societies still often goes unnoticed. Why do we celebrate climbing the highest mountains, yet overlook the exploration of the deepest caves? Why are so many unaware of the remarkable biodiversity thriving underground? And why do we study distant galaxies while Earth's subterranean environments may hold solutions to today's ecological and societal challenges?

Considering the importance of communicating these findings to inform real‐world decision‐making, this review aims to equip researchers and practitioners with a comprehensive *vade mecum* of examples, concepts, and ideas for conveying the importance of subterranean ecosystems. Effective communication requires tailoring messages to specific target audiences, using the right metaphors and psychological triggers. For some, subterranean biodiversity can be framed as a form of ‘life insurance’, emphasising its role in maintaining ecosystem stability and resilience (Loreau *et al*., 2021). Others may respond to economic metaphors, recognising the monetary value of services like water filtration, carbon sequestration, and raw material provision. At the same time, indigenous cultures, which have depended on subterranean ecosystems for centuries, offer invaluable traditional ecological knowledge and biocultural values that can enrich natural resource management strategies (Gibson *et al*., 2022) These perspectives often tap into metaphors related to the spiritual connection with these places, whereas the aesthetic allure and sense of mystery inherent to subterranean ecosystems can captivate audiences drawn to the unknown (Ryan‐Davis & Scalice, 2022).

By integrating these diverse perspectives, we can foster a deeper appreciation for subterranean ecosystems and their role in sustaining life on Earth. Subterranean biodiversity is not just a scientific curiosity, it is a cornerstone of planetary health, a source of resilience in the face of environmental change, and a testament to the interconnectedness of all ecosystems. Yet, we are in danger of losing subterranean biodiversity before we even have the chance to acknowledge its true value (Mammola *et al*., 2019). With this awareness, we can transform awkward questions about subterranean ecosystems into opportunities for inspiration and advocacy. As the world rallies to address environmental change and biodiversity loss, acknowledging and valuing the vital services provided by nature is essential to driving meaningful progress toward a more sustainable future. Ultimately, ensuring that subterranean ecosystems receive the attention and protection they deserve begins with one simple act: shifting the attitude of the next listener from indifference to appreciation.

## IX. CONCLUSIONS

(1) Interconnected terrestrial, freshwater, and marine subterranean environments, constituting one of the most widespread ecosystems on Earth, host globally significant biodiversity that plays a key role in maintaining a healthy biosphere. However, many of the ecological and societal contributions of subterranean ecosystems remain largely unquantified.
(2) By mapping the contributions of subterranean ecosystems onto the Common International Classification of Ecosystem Services (CICES Version 5.1), we found that they contribute to up to 75% (68 out of 90) of the ecosystem services identified by CICES. More specifically, subterranean ecosystems contribute to 63% of Provisioning services, 82% of Regulation & Maintenance services, and 100% of Cultural services.
(3) Key provisioning services include water (with 43% of irrigation and 49% of domestic water sourced from groundwater), food (e.g. groundwater supports most surface vegetation, including important crops like rice), and energy (primarily from the growing geothermal sector). Additionally, subterranean ecosystems are vital for material extraction (ranging from diverse minerals and rocks to bat guano) and represent a largely untapped source of biomolecular resources.
(4) Due to their extensive surface–subterranean interconnections, these ecosystems are central to global water and (bio)geochemical cycles, including those of carbon, nitrogen, and other key elements. They also play a critical role in regulating and maintaining various physical and chemical conditions, mitigating pollutants and greenhouse gases, and supporting essential biological processes across the entire surface–subterranean ecological gradient.
(5) Subterranean ecosystems also hold cultural significance. Tourist caves, for example, rank among the most visited geo‐ and ecotourism attractions worldwide. Subterranean ecosystems further inspire art and science, and are integral to the cultural and spiritual heritage of both contemporary and historical societies.
(6) Because subterranean ecosystems are largely hidden from view, their contributions are often overlooked. To address this, our review aims to equip researchers and practitioners with a comprehensive *vade mecum* of examples, concepts, and strategies to communicate the importance of subterranean ecosystems effectively. Importantly, impactful communication requires tailoring messages to specific audiences and using appropriate metaphors and psychological cues.

## AUTHOR CONTRIBUTIONS

S. Mam., C. G., and P. C. conceived the main idea, with suggestions by all authors. All authors contributed to the classification of services (Appendix S1). S. Mam. and A. Be. analysed the data. S. Mam. wrote the first draft. All authors contributed to the writing of specific sections and provided suggestions and additions to the overall text.

## CONFLICT OF INTEREST STATEMENT

None of the authors have a conflict of interest to disclose.

## Supporting information


**Appendix S1.** Mapping of subterranean ecosystem services using the Common International Classification of Ecosystem Services (CICES Version 5.1) (Haines‐Young & Potschin‐Young, 2018).

## Data Availability

Data supporting this study are available in Appendix S1. R code to reproduce the analysis is available in Github (https://github.com/StefanoMammola/Subterranean-ecosystem-services).
